# Finite Element Analysis of Orthopedic Hip Implant with Functionally Graded Bioinspired Lattice Structures

**DOI:** 10.3390/biomimetics5030044

**Published:** 2020-09-12

**Authors:** Nikolaos Kladovasilakis, Konstantinos Tsongas, Dimitrios Tzetzis

**Affiliations:** Digital Manufacturing and Materials Characterization Laboratory, School of Science and Technology, International Hellenic University, 14km Thessaloniki, 57001 N. Moudania, Greece; n.kladovasilakis@ihu.edu.gr (N.K.); k.tsongas@ihu.edu.gr (K.T.)

**Keywords:** topology optimization, TPMS structures, lattice structures, FEA, FEM, hip implant, trabecular bone, gyroid, Schwartz diamond, Voronoi

## Abstract

The topology optimization (TO) process has the objective to structurally optimize products in various industries, such as in biomechanical engineering. Additive manufacturing facilitates this procedure and enables the utility of advanced structures in order to achieve the optimal product design. Currently, orthopedic implants are fabricated from metal or metal alloys with totally solid structure to withstand the applied loads; nevertheless, such a practice reduces the compatibility with human tissues and increases the manufacturing cost as more feedstock material is needed. This article investigates the possibility of applying bioinspired lattice structures (cellular materials) in order to topologically optimize an orthopedic hip implant, made of Inconel 718 superalloy. Lattice structures enable topology optimization of an object by reducing its weight and increasing its porosity without compromising its mechanical behavior. Specifically, three different bioinspired advanced lattice structures were investigated through finite element analysis (FEA) under in vivo loading. Furthermore, the regions with lattice structure were optimized through functional gradation of the cellular material. Results have shown that optimal design of hip implant geometry, in terms of stress behavior, was achieved through functionally graded lattice structures and the hip implant is capable of withstanding up to two times the in vivo loads, suggesting that this design is a suitable and effective replacement for a solid implant.

## 1. Introduction

The additive manufacturing (AM) process has enabled the production of complex geometries [1,2,3,4] along with composite material structures [5,6], which are difficult to fabricate with traditional manufacturing techniques, such as machining and injection molding. This led to rapid development of the topology optimization process with advanced geometries via generative design and cellular materials. Topology optimization (TO) is a procedure that optimizes the material mass distribution within the already defined external volume [7], resulting in light-weight structures and minimization of feedstock material usage. The main objective is to maintain the desired mechanical properties reducing the mass of the structure; however, it also creates additional advantages such as high porosity and high surface-area-to-volume ratio [8]. There are two different approaches to achieve TO: the density-based and truss-based approaches. The density-based approach is identical to generative design [9]. On the other hand, the truss-based approach utilizes periodic unit cells (lattice structures) in order to achieve the optimum mass distribution [10]. Nowadays, there is a plethora of applications in several industries with topologically optimized products using both of the two aforementioned approaches, indicatively in the aeronautical, automotive, and biomechanical industries [11,12,13].

The truss-based approach imitates the cell structures from natural tissues, such as bones, corals, foams, and so forth. Thus, this approach is suitable for topology optimization of structures in biomechanical applications, such as implants and tissue scaffolds. Specifically, in this approach, the selected volume fills with lattice structures with specific geometry and dimensions replacing the solid material. Besides the advantage of minimizing the mass of the product for usage in biomechanical applications, this procedure offers the above-mentioned additional advantages, which facilitate the tissue regeneration process and allow diffusion of oxygen and nutrients [14,15]. There is a plethora of different lattice structures, the simplest ones being the 2D lattice structures like honeycombs and prismatic ones [16]. The geometrical complexity increases in 3D lattice structures, resulting in a vast number of diverse 3D lattice structures with further classification in strut structures and sheet structures [17]. The most widespread strut structures are the Octet and the Voronoi, which directly emerge from structures found in nature. On the other hand, the most common lattice sheet structures are the sheet triply periodic minimal surface (TPMS) structures such as Gyroid, Schwarz Diamond, and Neovius. According to Gibson et al. [18], all lattice structures lead to reduction of the mechanical properties. The reduction of the mechanical properties in lattice structures depends mainly on the relative density and on the actual geometry of the applied lattice structure. Relative densities less than 50% enhance the size effect, which has an increasing influence on the mechanical performance as the relative density is reduced. However, not all lattice structures have the same mechanical behavior; specifically, lattice structures that exhibit stretching-dominated behavior are less affected by the size effect compared to lattice structures that exhibit bending-dominated behavior [19]. Moreover, Al Ketan et al. [20] propose lattice structures that have stretching-dominated behavior for biomechanical applications which receive increased stresses.

In addition, there are several studies that have tried to combine additive manufacturing techniques with orthopedic implants through customization and lattice structures. Mahmoud et al. [21] have gathered the majority of these studies in a comprehensive review, summarizing the use of AM technologies to produce orthopedic implants from lattice structures and functionally graded materials. Gabbrielli et al. [22] and España et al. [23] investigated the possibility of using lattice structures in acetabular cups for mechanical and biological advantages. Moreover, Hazlehurst et al. [24] suggested manufacturing a hip implant consisting of cubic lattice structures in the whole internal body of the implant and this led to severe reduction of the implant’s stiffness and strength. Furthermore, Limmahakhun et al. [25] and González et al. [26] investigated innovative designs in order to optimize the utility of lattice in orthopedic implants and increase their mechanical strength and this was achieved by functional gradation of the lattice structures.

**Figure 1:**
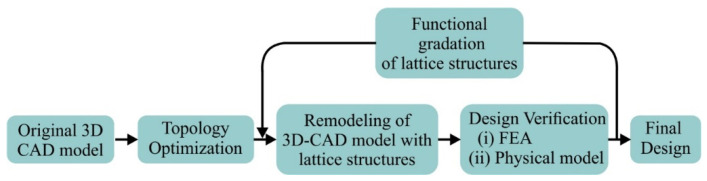
Flowchart of topological optimization procedure.

The aim of this research was to extract an innovative design for a bioinspired hip implant utilizing topology optimization tools. Therefore, the current paper proposes a novel hip implant design with advanced lattice structures in order to address the reduction in strength and handle the in vivo loadings. Figure 1 portrays a flowchart of the topology optimization process through lattice structures depending on the mechanical behavior. Initially, a hip implant was designed according to international medical standards and then its mechanical behavior was examined under in vivo static loads through finite element analysis (FEA). Furthermore, specific regions of the implant, where topology optimization was essential, were replaced by bioinspired lattice structures that have been shown to exhibit stretching-dominated behavior, such as the Voronoi strut structure and the Gyroid and Schwarz Diamond sheet structures. These TPMS structures were selected due to the promising mechanical strength that was exhibited in the aforementioned studies [19,20]. In addition, Voronoi structure was selected because it displays similar behavior to the trabecular internal structure, while demonstrating high mechanical strength [27,28]. These three lattice structures were examined and evaluated for their mechanical performance under the same in vivo loads. In addition, further design optimization was applied to lattice structures through functional gradation in order to improve the mechanical properties of a hip implant.

## 2. Materials and Methods

### 2.1. Design of Hip Implant

Hip implant design follows a series of international standards in order to fulfill its purpose [29]. A typical orthopedic hip implant consists of three distinct regions: head, neck, and intramedullary stem (rest of the body). The main design considerations are the head diameter, the diameter and length of the neck, the length of the intramedullary implant (stem), the stem cross-section area, and the angle of placement of the head relative to the main body. 

Design parameters are configured from past research and existing literature. According to the studies of Charnley et al. [30] and McKnee et al. [31], the head diameter ranges from 22 mm to 45 mm, in order to fit in the hip joint. Moreover, the length and diameter of the neck range from 10 mm (short-neck) to 40 mm (long-neck) and 13 mm to 30 mm, respectively [32,33]. The length of the intramedullary stem of the hip implant ranges from 120 mm to 180 mm and the neck-shaft angle varies between 135° and 145° [34]. Table 1 summarizes the design parameters and their aforementioned ranges.

**Figure 2:**
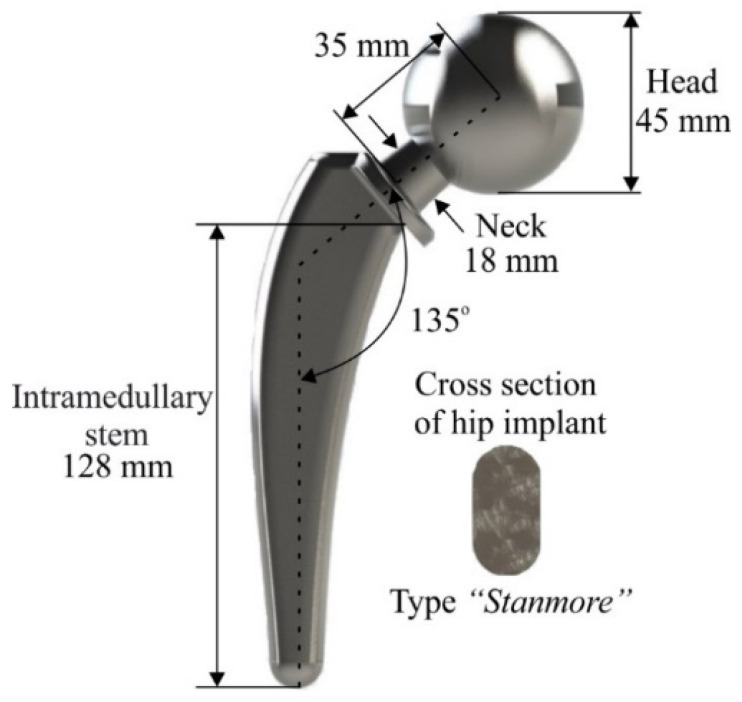
Design of hip implant.

In order to apply topology optimization through lattice structures on an orthopedic hip implant, a new hip implant was designed fulfilling all the above-mentioned standards. Therefore, the designed hip implant has the following geometry and dimensions. More specifically, the intramedullary stem’s length is 128 mm, while the neck’s length is 35 mm. In addition, the diameter of the neck is 18 mm and the diameter of the head is 45 mm. The placement angle of the neck and head in relation to the axis of the implant is 135° degrees and the angular range is almost 120° degrees. Figure 2 portrays the design of the hip implant, its basic dimensions, and its cross-section, as it was designed in SolidWorks™ software.

### 2.2. Lattice Structure Configuration

Cellular materials are scattered in naturally formed structures, such as foams, corals, bones, and so forth. These natural materials are the result of the evolution of thousands of years, thus both mechanical properties and functional properties have been optimized depending on each application. There is a variety of lattice structures that differ in shape and geometry. Therefore, for the topology optimization of a human body implant, it is logical to investigate lattice structures derived from nature that could serve the function of the implant.

**Figure 3:**
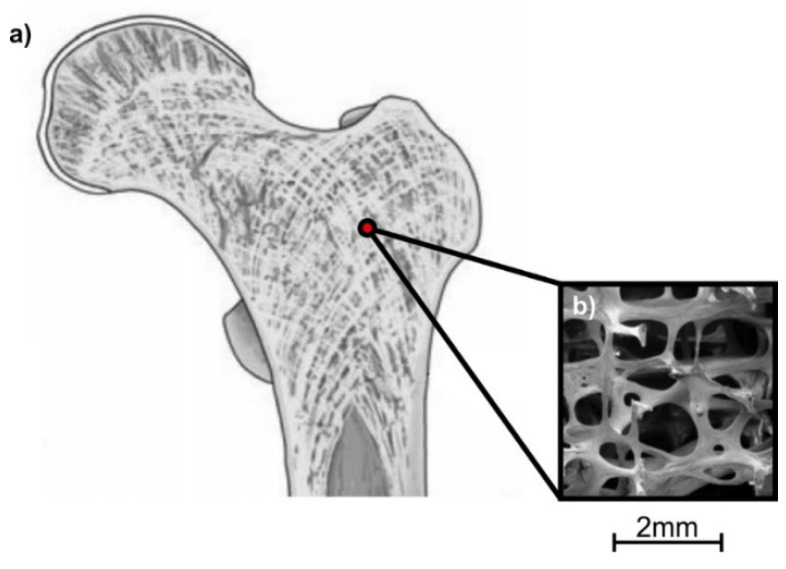
Trabecular bone: (**a**) trabecular bone schematic [36]; (**b**) trabecular/Voronoi-like structure examined by scanning electron microscope (SEM) [37].

In the case of hip implant, lattice structures should be able to withstand increased loads relative to the body weight and the human movement. Hence, a potential candidate structure which imitates the inside trabecular structure is the Voronoi truss structure [33,34]. 3D Voronoi structures follow this mathematical formula [35]:

*V*(p*_i_*) = {p/d(p,p*_i_*) ≤ d(p,p*_j_*), *j* ≠ *i*, *i*, *j* = 1, ..., *n*} (1)

where p_1_, …, p*_n_* are the distinct seeds in 3D space, d (p,p*_i_*) is the Euclidean distance between two seeds (p and p*_i_*), and *V*(p*_i_*) represents the 3D Voronoi polygon on seed p*_i_*. Figure 3 shows the structure of trabecular bone and an indicative Voronoi/trabecular-like structure.

**Figure 4:**
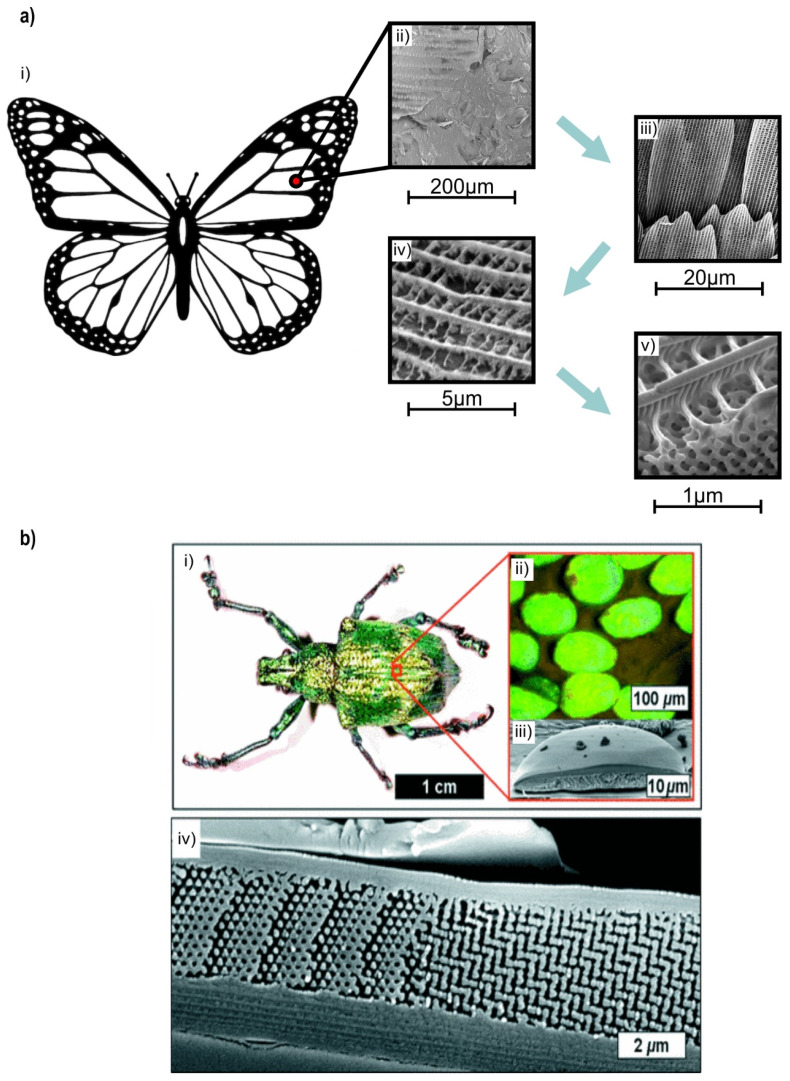
TPMS-like structures found in nature: (**a**) Gyroid in butterfly wings: (i)butterfly schematic was retrieved from [42]; (ii-v) SEM micrographs on a cross sectional view of a butterfly wing scale, where (v) was reproduced from [38]; (**b**) Schwarz Diamond in L. Augustus beetle shell (i) Photograph of the weevil L. Augustus. (ii) Optical micrograph of individual scales attached to the exoskeleton of L. Augustus under white-light illumination. (iii) Cross-sectional SEM image of a single scale. (iv) Detailed cross-sectional SEM image of a region of a scale. (Reproduced from [40], with permission of APS).

Other bioinspired lattice structures that could withstand high stress loads and offer the proper conditions to facilitate tissue regeneration are triply periodic minimal surfaces (TPMSs). TPMSs are characterized by surfaces that have an average curvature value equal to zero. TPMS-like geometries result from applying Fourier transformations on level-set equations and by using a level-set parameter c in Equations (2) and (3) [15]. According to the literature [16], the most advanced TPMS structures, with increased strength, are the Gyroid and Schwarz Diamond structures. A Gyroid-like structure is also observed on butterfly wings (Figure 4a), offering high tolerance in bending loads [38]. A Schwarz Diamond-like structure is used by beetle L. Augustus in order to reinforce its shell structure (Figure 4b) and has also shown remarkable mechanical strength in various experiments [39,40,41]. It is worth noting that in this study, the aforementioned structures have been used for the topology optimization of the hip implant. The design software nTopology™ was also used to design and implement these structures in hip implant geometry. The level-set equations for Gyroid-like and Schwarz Diamond-like geometries are shown in the following equations:

φ_g_ = sin *x* cos *y* + sin *y* cos *z* + sin *z* cos *x* = c (2)

φ_d_ = sin *x* sin *y* sin *z* + sin *x* cos *y* cos *z* + cos *x* sin *y* cos *z* + cos *x* cos *y* cos *z* = c (3)

### 2.3. Finite Element Analysis

Finite element analysis (FEA) simulated each hip implant design with different lattice structures under static in vivo conditions. It is worth mentioning that FEA for each implant focused mainly on the determination of yield point and the calculation of safety factor of the topologically optimized implant design. ANSYS™ software was used for the finite element analysis and in particular, the static structural module was used to simulate the quasi-static loading. Generally, mesh generation for lattice structures could be implemented with two different types of elements: hexahedrals [43] and tetrahedrals [44]; in these studies, plenty of references are given towards the use of the different discretization methods. In this research, the mesh consisted of tetrahedral elements for the whole body of the implant. In the last decade, a new material was proposed for medical use and especially for orthopedic implants; this novel material is the nickel-based superalloy Inconel 718. According to the literature [45], Inconel 718 offers enhanced corrosion resistance and advanced mechanical properties. However, in order to be suitable for this application, Inconel should fulfill the international standards of ASTM F90-14 and UNS N07718 [46]. Table 2 and Table 3 present the chemical composition and the mechanical properties of Inconel 718 which were used in order to build a material model combining isotropic elasticity and bilinear isotropic hardening.

Furthermore, in order to complete the FEA model, it is necessary to determine, based on the literature, the conditions for the implant’s support and the maximum loads that the implant receives from the human body under in vivo loading conditions. Hence, according to Ducheyne et al. [47], the hip implant has fixed support at the bottom of its structure in contact with the femur bone, without relying on calcar support. The received loads remain to be defined as to the point of application, the direction of the loading, and the amount of forces. El-Shiekh et al. [28], who have carried out a comprehensive research in hip joint replacement under static and dynamic loading, have shown that the forces caused by the human body’s weight are applied on the center of the hip implant’s head. McLeish et al. [48] concluded on the results that for zero degree of pelvis angle, the direction of the human body’s force is approximately at 20° degrees to the center axis of the femur bone. Finally, the magnitude of the force depends on the weight of the human body and the type of body movement. Bergmann et al. [49] proposed a percentage relationship between the type of movement and the weight of the human body. The percentage weight relations, depending on the type of movement, are listed in Table 4 as well as the nominal values of the force corresponding to an average body weight of 75 kg [50].

## 3. Results and Discussion

### 3.1. Finite Element Analysis for Solid Hip Implant

**Figure 5:**
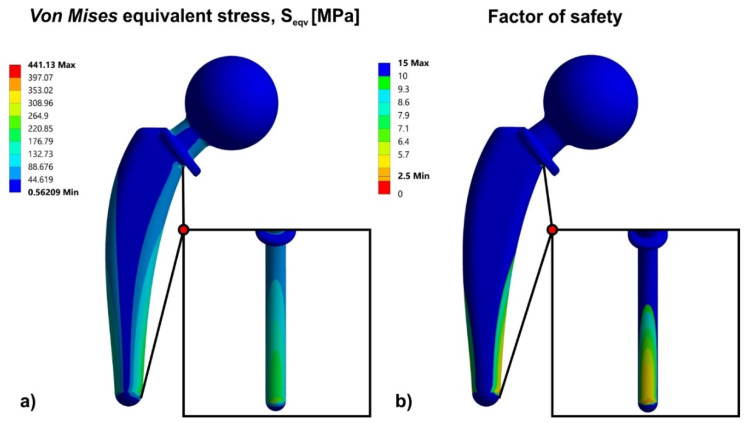
(**a**) Von Mises stress contour; (**b**) factor of safety (FOS) contour.

Before the topology optimization process, it was necessary to evaluate the initially designed solid hip implant’s mechanical behavior through finite element analysis. Therefore, the 3D CAD model of the solid hip implant (Figure 2) was examined with FEM under in vivo conditions, as described previously. Figure 5 shows the contours of von Mises stresses (Figure 5a) and the contours of the factor of safety (Figure 5b) for maximum load (i.e., at a force of 5300 N). These analyses extracted a series of conclusions which are essential for the topology optimization process. The first conclusion is that the head (ball) where the loads have been applied has shown low stress concentration, hence it obtains small strains compared to the rest of the body. It was expected that the maximum stress would occur in a region adjacent to the support of the hip implant. This is because the static system works like a cantilever beam and the maximum bending moment will occur at the fixed boundary condition. Therefore, both the neck and the intramedullary stem are divided into three distinct regions (Figure 5) of strain: the tensile region (left side), the low stresses region (center), and the compressive region (right side). In the tensile region, due to the direction of the applied force, tensile stresses were concentrated, however, the amount of stress was low, revealing that this region has moderate risk for fracture. The central region of implant was also the area with the lowest stress concentration, where the improvement of mass distribution is essential. Moreover, the compressive region is the area where, due to the direction of the applied force, there is concentration of compressive stresses and intervention is needed during the process of topology optimization. 

As shown in Figure 5, the maximum stresses were compressive and they reached up to 441 MPa. However, these stresses occurred only in one specific point and did not reflect the total mechanical behavior of the implant, as the rest of the stresses were in the range between 90 MPa and 300 MPa. Nonetheless, even the maximum stresses were much lower than the material yield point. This was deducted since the factor of safety (FOS) has shown a minimum value of 2.5. Therefore, it could be concluded that the hip implant with this specific material and design withstands loads 2.5 times more than the maximum load that the implant receives from the human body in tripping movement, thus an extensive topology optimization of the design could be performed.

### 3.2. Topology Optimization through Lattice Structures

The process of topology optimization of the hip implant’s design has been analyzed using three different lattice structures, namely, the Voronoi strut structure and the Gyroid and the Schwarz Diamond sheet structures, which are bioinspired. The objective was to evaluate the mechanical behavior of each structure and to highlight the most advanced structures in order to proceed to the next phase of optimization, which was the adaptive adjustment of the lattice structure through functional gradation. Topology optimization exploits the findings of the FEA of the solid hip implant in order to define certain areas of the design to be optimized. The next step was to select the appropriate relative density of the proposed lattice structures to be applied in these regions. The appropriate value of the relative density should be defined by the right trade-off between the deterioration of the mechanical properties, due to size effect, and the desired advantages (i.e., reduction of the mass and increased porosity). Therefore, some of the drawbacks of the size effect, like reduced modulus or a loss in strength, could be overcompensated by lightweight structures with enhanced porosity. Thus, the relative density was chosen to be 50%, due to the fact that it has a negligible influence from the size effect and it was close enough to the relative density of the femur bone [51] enhancing the body’s ability to regenerate tissues.

**Figure 6:**
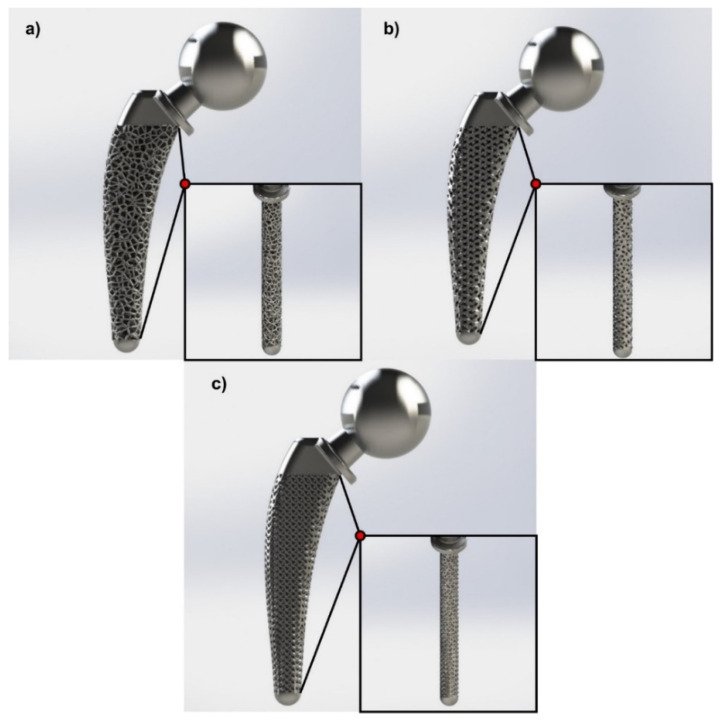
Optimized hip implant designs via lattice structures: (**a**) Voronoi; (**b**) Gyroid; (**c**) Schwarz Diamond.

The selection of redesigned regions with lattice structure was a result of FEA in the solid hip implant. Therefore, as illustrated in Figure 5, due to increased loads, both compressive and tensile in the neck of the implant, the whole area remained solid. The loads of the implant were applied on the head; thus, it was necessary to have a continuous surface of 2 mm thickness. Since the implant’s head demonstrated low stress concentration, only its interior was redesigned with lattice structures, as shown in Figure 6. In addition, Figure 6 shows that the whole intramedullary stem was redesigned with lattice structures, except the region where the implant was mounted, where it remained solid. Figure 6a–c portrays two different views, side and front, of the Voronoi, Gyroid, and Schwarz Diamond structures, respectively.

**Figure 7:**
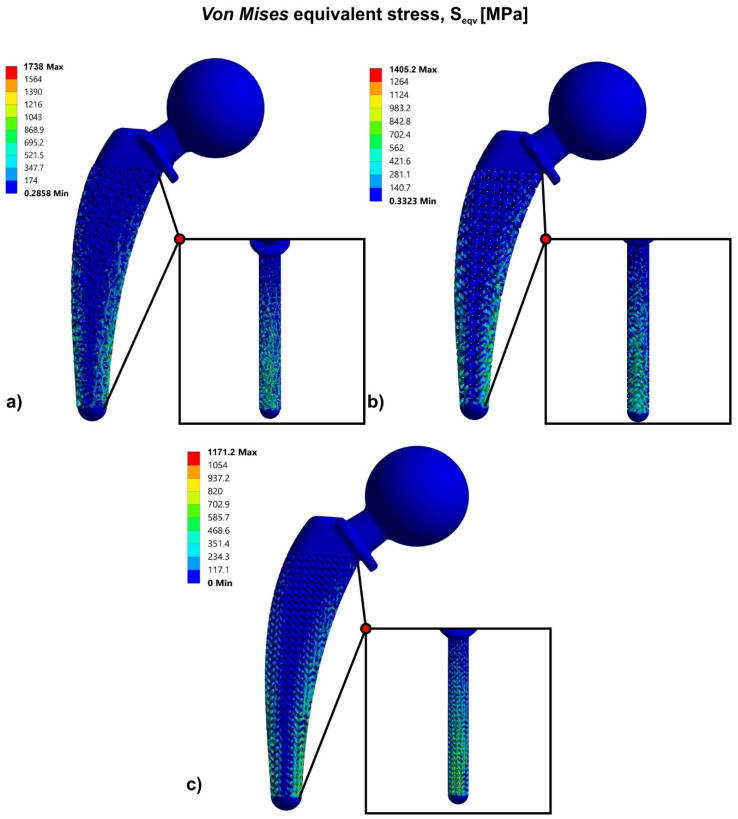
Von Mises stress contours for: (**a**) Voronoi structure; (**b**) Gyroid structure; (**c**) Schwarz Diamond structure.

Figure 7 portrays the results of the finite element analysis for three different approaches of the hip implant’s topology optimization, using bioinspired lattice structures such as Voronoi, Gyroid, and Schwarz Diamond structures. Specifically, according to the FEA results of the solid hip implant, the topology optimization approach has shown that the highest strength was observed in the hip implant that contained the Schwarz Diamond structure. The hip implant which contained Schwarz Diamond portrayed the lowest stress concentration with a maximum value of 1171 MPa at the compressive region. In addition, the hip implant with Gyroid structure revealed ultimate compressive stresses at a value of 1405 MPa. The hip implant with Voronoi structure has demonstrated the weakest behavior compared to the other structures under study, with maximum stress concentration at 1738 MPa.

Τhe factor of safety was calculated by the maximum stress concentration of each structure, as listed in Table 5. In addition to the standard FOS, FOS_Ult_ has been also used and it was calculated as the ultimate factor of safety which depends on the ultimate strength of the material, as shown in Table 5. As the FOS values verified, only the Schwarz Diamond structure could withstand the forces of the human body without fracture of the implant, as FOS_Ult_ was greater than the value of 1 (FOS_Ult_ =1.17 > 1). However, the stress concentration in the Gyroid structure was marginally greater than the fracture point of the material, thus it is expected that with an improved design approach through functional gradation, the strength of the implant could be increased. On the other hand, the Voronoi structures should exhibit a fundamental design modification in order to achieve FOS values greater than 1.

### 3.3. Functional Gradation of Lattice Structures

**Figure 8:**
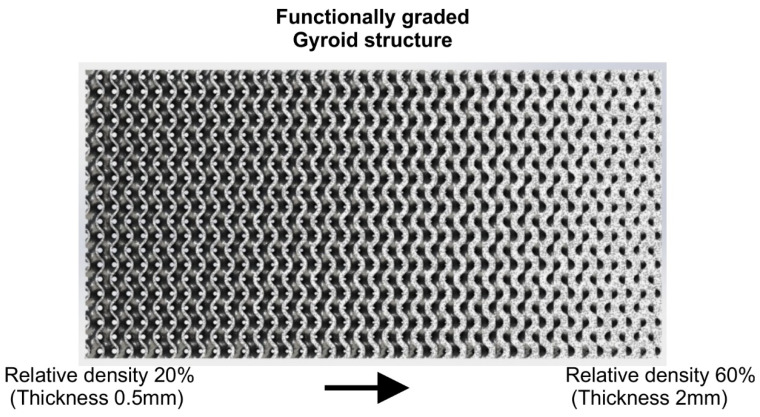
Graphical illustration of functionally graded Gyroid structure.

Finite element analyses of the topologically optimized versions of the hip implant through bioinspired lattice structures showed the mechanical superiority of sheet structures, and especially TPMS, over strut structures, as discussed previously. However, through functional gradation of lattice structures, it is possible to achieve further topology optimization of the part. Thus, knowing the regions where the stresses are concentrated, design could be changed to increase or decrease the local relative density of the selected lattice structures, keeping the total relative density of the object constant but increasing its mechanical performance. Figure 8 illustrates a graphical representation of a functionally graded Gyroid structure with mean relative density of 40%.

**Figure 9:**
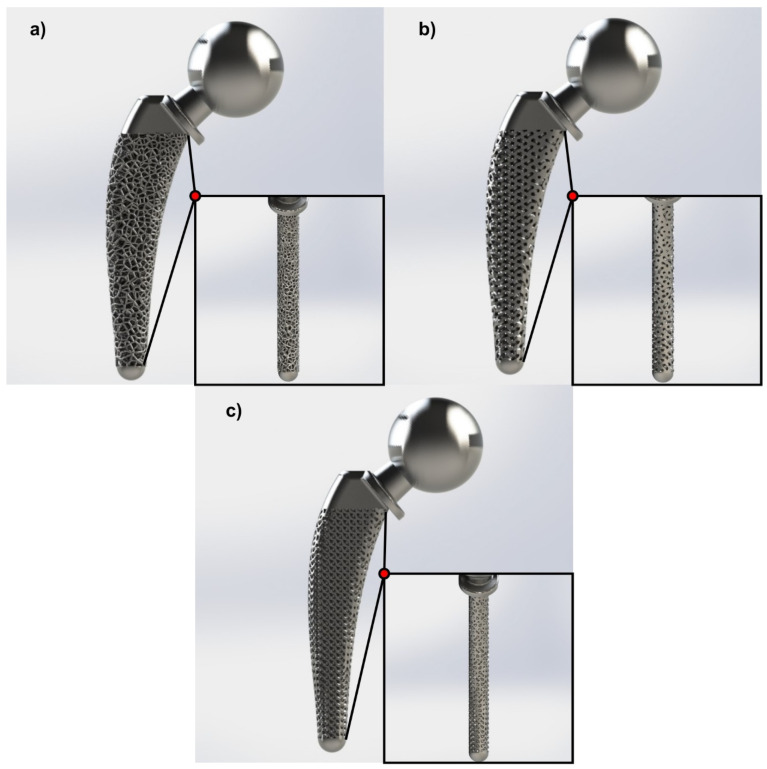
Functionally graded hip implants for lattice structures: (**a**) Voronoi; (**b**) Gyroid; (**c**) Schwarz Diamond.

This procedure was followed for all above-mentioned structures. In the areas where the highest stresses (compressive region) were concentrated, it was decided to apply a denser pattern of lattice structure to reach a local relative density of 65%, in order to completely eliminate the size effect. On the other hand, in the region with low stresses (i.e., at the center of the implant), a sparser local relative density was decided to be implemented as the stresses in this area were lower, hence the integrity of the object would not be affected by the size effect. Since the aim was to maintain the total relative density constant, these two areas were separated using a try-and-error algorithm, in such a way as to produce redesigned hip implants that have the same mass as the Voronoi, Gyroid, and Schwarz Diamond structures. It is worth mentioning that the topology optimization of the hip implant’s head and the tensile region remained the same as the previous design, as further removal of material may have affected locally the integrity of the object’s mechanical behavior. Figure 9 shows the final geometries of the topologically optimized hip implant with Voronoi structure (Figure 9a), hip implant with Gyroid structure (Figure 9b), and hip implant with Schwarz Diamond structure (Figure 9c). All the hip implant designs with functional gradation of lattice structures were designed and calculated using the nTopology™ platform.

**Figure 10:**
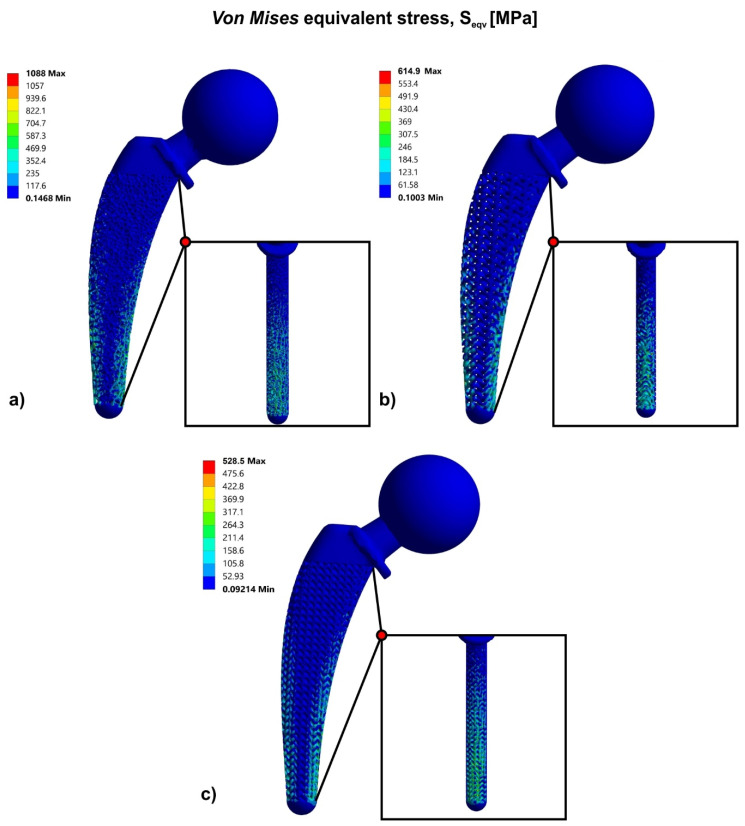
Von Mises stress contours of functionally graded hip implant with: (**a**) Voronoi structure; (**b**) Gyroid structure; (**c**) Schwarz Diamond structure.

Figure 10 illustrates the results of FEA and the respective contour of stresses for the hip implant with Voronoi structure (Figure 10a), with Gyroid structure (Figure 10b), and with Schwarz Diamond structure (Figure 10c). Figure 10a shows that the topologically optimized hip implant with functional gradation of Voronoi structure concentrated lower stresses than the corresponding design approach with Voronoi without functional gradation. Specifically, this hip implant design has shown a peak of compressive stresses at 1088 MPa.

Furthermore, the topologically optimized hip implant with functional gradation of Gyroid and Schwarz Diamond structures revealed even more lower compressive stresses which resulted in a remarkable improvement of the hip implant’s mechanical performance. As it is shown in Figure 10b,c, these hip implant designs have demonstrated a maximum value of compressive stress at 615 MPa and 529 MPa, respectively.

In order for the redesigned hip implants to handle the forces that they receive from the human body, it is important to access their mechanical behavior through the factor of safety. Hence, the factor of safety for the design approach containing the lattice structure of functionally graded Voronoi was 1.01 and for the design approach containing the TPMS structures of functionally graded Gyroid and Schwarz Diamond were 1.79 and 2.08, respectively. These results showed that the design approaches of the hip implant which consisted of functionally graded lattice structures could withstand the maximum static loads that the hip joint receives under in vivo conditions. Regarding the functionally graded Voronoi structure hip implant, it could marginally withstand these loads without being plastically deformed, as shown in the values of FOS in Table 6. The functionally graded TPMS structures, Gyroid and Schwarz Diamond, could handle almost twice the loads (Table 6).

## 4. Conclusions

In this paper, an orthopedic hip implant was designed according to international standards and then a topology optimization of its geometry was performed. The topology optimization process was implemented via bioinspired lattice structures, namely, Voronoi, Gyroid, and Schwarz Diamond structures, which are derived from nature having superior mechanical performance. Moreover, topology optimization occurred with the implementation of these lattice structures in regions of low stress, in order to achieve the optimal mass distribution within the existing volume. Furthermore, functional gradation of the implemented lattice structures was performed. In particular, it was observed that the hip implant that contained Schwarz Diamond structures revealed the best mechanical behavior, both in simple topology optimized implant and in functionally graded implant. The factor of safety of the functionally graded Voronoi was 1.01 and for the design approach containing the TPMS structures of functionally graded Gyroid and Schwarz Diamond were 1.79 and 2.08, respectively. Topology optimization leads to a reduction of weight of 38% compared to the solid version of the hip implant, therefore less construction material is needed. Moreover, for the intramedullary stem, it has a 50% mean porosity to facilitate the process of tissue regeneration through diffusion of cells, oxygen, and other nutrients. Future work will focus on the fabrication of these implants, utilizing additive manufacturing methods and the evaluation of their mechanical properties will be measured.

## Figures and Tables

**Table 1:** Typical ranges for hip implant’s design parameters.

Design Parameters	Typical Values
Length of intramedullary stem	120 mm–180 mm
Length of neck	10 mm–40 mm
Head diameter	22 mm–45 mm
Neck diameter	13 mm–30 mm
Angle of head placement	135°–145°

**Table 2:** Chemical composition of Inconel 718 (UNS N07718).

Chemical Requirements							
	**Ni**	**Fe**	**Mo**	**Mn**	**Si**	**Cr**	**C**
**Min (%)**	50	Balance	2.8	0.35	0.35	17	0.08
**Max (%)**	55		3.3			21	

**Table 3:** Mechanical Properties of Inconel 718 (UNS N07718).

Mechanical Properties	Typical Values
Density	8.19 g/cm^3^
Elastic Modulus	200 GPa
Poisson Ratio	0.29
Yield Strength	1100 MPa
Ultimate Yield Strength	1375 MPa

**Table 4:** Loading of hip implant for different types of movement.

Type of Movement	Max. Load (% Weight)	Max. Force on Hip Joint
Slow walking	282	2075 N
Climbing upstairs	356	2620 N
Climbing downstairs	387	2850 N
Tripping	720	5300 N

**Table 5:** Factor of safety for three different topology optimization approaches.

Lattice Structure	Factor of Safety (FOS)	Ultimate Factor of Safety (FOS_Ult_)
Voronoi	0.63	0.79
Gyroid	0.78	0.98
Schwarz Diamond	0.94	1.17

**Table 6:** Factor of safety for all examined versions of hip implant’s design.

Hip Implant Versions	Factor of Safety (FOS)	Ult. Factor of Safety (FOS_Ult_)	Weight (g)
Solid	2.5	3.12	975
Topology optimized			
Voronoi	0.63	0.79	600
Gyroid	0.78	0.98	
Schwarz Diamond	0.94	1.17	
Topology optimized and functionally graded			
Voronoi	1.01	1.26	600
Gyroid	1.79	2.24	
Schwarz Diamond	2.08	2.6	
